# Validation of a Simplified Digital Periodontal Health Screening Module for General Dental Practitioners

**DOI:** 10.3390/healthcare10101916

**Published:** 2022-09-30

**Authors:** Shahida Mohd-Said, Nur Adila Mohd-Norwir, Ahmad Najmi Ariffin, Huey Shiuan Teo, Nik Madihah Nik-Azis, Haslina Rani, Haslinda Ramli, Juzaily Husain, Tuti Ningseh Mohd-Dom, Afendi Hamat

**Affiliations:** 1 Department of Restorative Dentistry, Faculty of Dentistry, Universiti Kebangsaan Malaysia, Kuala Lumpur 50300, Malaysia; 2 Faculty of Dentistry, Universiti Kebangsaan Malaysia, Kuala Lumpur 50300, Malaysia; 3 Klinik Pergigian Batu Pahat, Klinik Kesihatan Batu Pahat, Jalan Kluang, Batu Pahat 83000, Malaysia; 4 Department of Family Oral Health, Universiti Kebangsaan Malaysia, Kuala Lumpur 50300, Malaysia; 5 Department of Periodontology and Community Oral Health, Faculty of Dentistry, Universiti Sains Islam Malaysia, Kuala Lumpur 55100, Malaysia; 6 Department of Restorative Dentistry, Kulliyah of Dentistry, International Islamic University Malaysia, Kuantan 25200, Malaysia; 7 Center for Research in Language and Linguistics, Faculty of Social Sciences and Humanities, Universiti Kebangsaan Malaysia, Bangi 43600, Malaysia

**Keywords:** dental health services, digital dentistry, periodontal disease, dental checkup

## Abstract

As a silent disease, individuals at risk of periodontitis are not easily identified until the disease has become severe. Early detection at the community level is essential, especially for general dental practitioners. The aim of this study was to design a comprehensive, user-friendly tool to screen patients’ periodontal health at community level and to evaluate users’ acceptance of its use. The periodontal health screening module was first developed by an expert panel of periodontists, public health specialists and general dentists. The developed module was tested for content acceptance on 156 graduating dental students from three public schools and later validated by 12 private general dental practitioners (GDPs) for reliability. Most of the students (64.1%) found the new module an easy assessment tool for periodontal health compared to the Basic Periodontal Examination (BPE). Most claimed that they understand the contents (80.8%) and accepted the designs (86.6%) and agreed (82.7%) that the new assessment module would allow them to screen patients anytime in the clinic. The interrater reliability as assessed between the GDPs and the investigators revealed acceptable agreement ranging from 62.5–100.0% (mean 89.6 ± 10.2%). The simplified digital periodontal health screening module showed promising acceptance for application in private general dental clinics.

## 1. Introduction

Periodontitis is a chronic inflammatory disease that is bacterial-induced and modified by the host’s immune and inflammatory response. The screening of periodontitis at the epidemiological level is essential as it could prevent the progression of the disease and prevent all the costly health complications [1]. Early detection of periodontal disease can result in the success of dental treatment and better prognosis and at the same time prevent the progression of the periodontal disease [2,3]. It is therefore important for all dental professionals, especially general dental practitioners (GDPs), to best identify individuals at risk and those who are not aware of their existing and ongoing periodontal disease, preferably as early as possible, for risk and signs of periodontitis and perform the necessary intervention including referral to the dental specialists for further management. Unfortunately, dentists tend to be less vigorous with the periodontal assessment [4] and the problem may often be picked up when it is at its worst, or in its severe form. This behaviour may have been caused by the limited chairside time that the GDPs have while seeing the patient [5], inadequate awareness of periodontal screening and or management, or due to the lack of confidence to do so.

The most well-known screening tool available for periodontal problems is the Basic Periodontal Examination (BPE). The BPE is a simple and rapid screening tool used to indicate the level of further examination needed and provide basic guidance on the treatment needed. These BPE guidelines are not prescriptive but represent a minimum standard of care for initial periodontal assessment [6]. However, the BPE is not a diagnostic tool and does not allow for full assessment and consideration of a patient’s medical and dental history [7,8]. Alternatively, a full periodontal evaluation will take up longer chairside time, which can be inconvenient and time-consuming for the GDPs in a busy daily practice. Hence, the development of a new periodontitis identification tool seems appropriate for ease of identification of periodontitis patients in dental practices.

Based on the recent 2017 periodontal and peri-implant diseases classification [9], a case is considered periodontitis in the context of clinical care if: Interdental clinical attachment loss (CAL) is detectable in two or more non-adjacent teeth, or buccal or oral CAL ≥3 mm with pocketing >3 mm is detectable in two or more teeth [10]. This inclusion should be considered when developing a new periodontal screening module in combination with a history-taking assessment. In addition, periodontal disease is critically evaluated based on an extensive full-mouth periodontal health examination which is a highly accurate diagnostic tool; however, it is time-consuming and costly.

Therefore, the development of an easy, quick, relatively accurate, low cost, and evidence-based periodontal health screening tool would be valuable to facilitate population-based studies and allow effective screening by dental clinicians in their practice at any time. The aim of this study was to design a user-friendly tool in the form of a comprehensive module to screen patients’ periodontal health and to evaluate users’ acceptance of its use. Findings from this study would be able to provide a clear idea for future efforts to strengthen strategies for detecting periodontal disease at an early stage and for intervention especially among those with higher risk and susceptibility to the disease.

## 2. Materials and Methods

This was a prospective study that involved two components: (1) the development of a simplified periodontal health screening module, and (2) the content validation of the module. Ethical approval was granted by the Research and Ethics Committee (reference: PPI/111/8/JEP-2020-617). Permission to conduct this study was granted by the Institutional Review Board.

### 2.1. Phase 1: Development of a Simplified Periodontal Health Screening Module

Panellists of three periodontists, two dental public health specialists, two periodontics specialist trainees, and a general dentist were selected to be involved in the development of this module. An online self-guided version of the periodontal health screening module was developed based on the British Society of Periodontology’s Basic Periodontal Examination (BSP 2019) guidelines, the Periodontal Risk Assessment (PRA) based on Lang and Tonetti’s [11] risk categories, periodontitis self-assessment [12], the Malaysian Association of Dental Public Health Specialist-Colgate Palmolive Malaysia kit (MADPHS-CP 2016), and the 2017 Classification of Periodontal and Implant Diseases [9].

**Figure 1:**
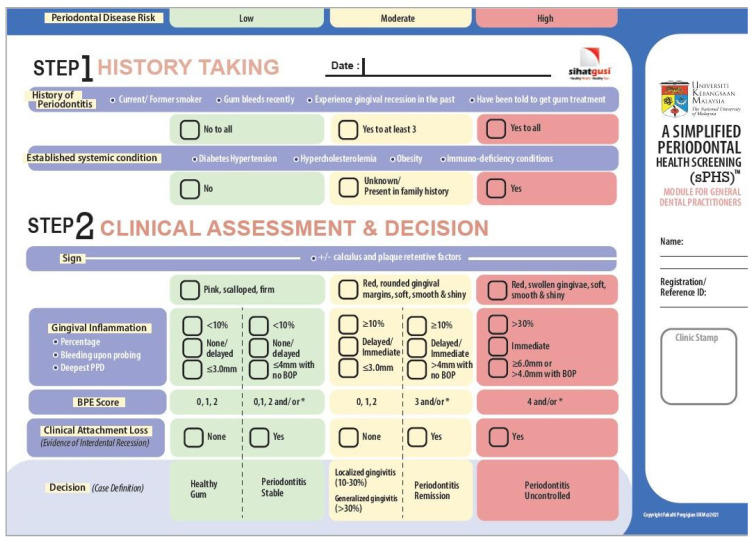
The finalised simplified Periodontal Health Screening Module.

The newly developed module consists of three parts: (i) history takings, (ii) clinical assessment, and (iii) case decision. The parts are further described in Supplementary S1. There are three columns and eight rows that require assessment from the participating practitioner. The row that has the highest number of ticks will be chosen as the case definition for the patient (See Figure 1).

### 2.2. Phase 2a: Content Validation of the Periodontal Health Screening Module

A case scenario with details of three different patients was given to dental undergraduate students in three public universities (see Supplementary S1 for details of the cases). The students were first instructed to use the BPE for the case definition of the case scenario as they are trained and familiar with its use in their clinic. Subsequently, the same case scenario was given again, and the students were instructed to use the newly developed module. Lastly, the students were asked to provide their feedback on an online form on their: (1) understanding of the module content, (2) feedback on the design of the module, and (3) perceptions on how the module would benefit their practice.

### 2.3. Phase 2b: Validation among Private Dental Practitioners (GDPs)

The inclusion criteria for the GDPs involved in this phase of the study were: (i) Registered with the local Dental Council and have a valid Annual Practising Certificate-verified through the Dental Council website; (ii) consented and willing to fully participate in the research conduct; (iii) literate in the Malay and English languages; and (iv) have a minimum working experience of two years following graduation.

The GDPs were invited to join the study through convenience sampling. All dentists were briefed and trained in one-on-one sessions using a training module with the case scenario that was given to the undergraduate students in the earlier phase. Upon completion of the training, each dentist performed a periodontal health assessment using the module on a minimum of two patients in their own dental practice. The patients were recruited randomly from their own patient list to mimic the day-to-day circumstances of periodontal screening in a dental practice. A designated principal investigator then performed the same assessment on the same patients as the gold standard. A comparison between the assessments made by the GDPs and the principal investigator was then performed.

### 2.4. Data Analysis

Descriptive analysis was used to describe the characteristics of the study participants. Agreement on the answers was calculated and reported as a percentage of accuracy (Inter-rater reliability, IRR). Data analysis was conducted using the Statistical Package for the Social Sciences (SPSS) version 26 (IBM Corp., Armonk, NY, USA) statistics software.

## 3. Results

### 3.1. Validation among Graduating Dental Students

#### 3.1.1. Acceptance among the Dental Undergraduates

A total of 156 final-year dental students, aged between 22 to 27 (mean of 23.5) from three public schools, participated in this part of the study with the majority (72.4%) being females. Most of the undergraduate trainees (67.9%) preferred Basic Periodontal Examination (BPE) as their current periodontal health assessment tool while a quarter chose Community Periodontal Index and Treatment Needs (CPITN) (Table 1).

#### 3.1.2. Perceived Competency when Applying the New Module

Based on the case scenario given, 70.5% of the trainees agreed that diagnosing patients would be easier using BPE but only 21.8% of the trainees found it easy to use BPE while managing patients (Table 2).

On the other hand, most of the trainees (64.1%) found the new module to be an easier assessment tool for periodontal health (Table 3). Most claimed that they understand the contents (80.8%) and designs (86.6%) well, and either strongly agreed or agreed (82.7%) that the new assessment module allows them to screen patients anytime in the clinic. In addition, almost three-quarters of the trainees (72.4%) strongly agreed or agreed that the new assessment module could help them to screen patients faster, and most (90.4%) were able to think better and make faster decisions while managing patients when applying the new module.

### 3.2. Validation among GDPs in the Private Sector

A total of 12 GDPs were invited and consented to participate in this study. The GDPs, whose ages ranged from 27 to 52 years old (μ, 36.17), were mostly female (9, 75%). All participating GDPs have been practising dentistry for at least more than four years (μ, 11.67) and most were of Malay ethnicity (10, 83.3%) (Table 4). The average time taken to complete periodontal screening on a patient using the newly developed module was 7 min (range: 5–11).

The Inter Rate Reliability (IRR) was calculated based on two patients for each GDP. IRR was counted based on several total matched answers between the GDPs and the principal investigator. Calculation of IRR in this study revealed percentage agreement ranging from 62.5% to 100.0%, which was in the acceptable range (Table 5). All case definitions were the same between the GDPs and the principal investigator except for patient number 16 (which was identified by Dentist H). The periodontal status of the patients selected by the GDPS were 13 Periodontitis Uncontrolled, 7 Periodontitis Stable, and 4 Periodontitis Remission.

## 4. Discussions

Several methods, including the BPE, CPITN, and PSR, are available to assist dentists in assessing and detecting periodontal disease during routine dental examinations. The participants in this study mostly reported using BPE and CPITN as periodontal screening methods, depending on their personal preferences and training, similar to other studies on periodontal screening [12,13,14]. Nearly all tools used for periodontal health screening assess the most severe pocket depth but no further information about the loss of interproximal attachment is assessed. Moreover, these tools cannot be used to diagnose periodontal disease. Instead, a full 6-point pocket examination with the recording of several clinical information is needed. This can be challenging for dental practitioners during a routine dental check-up.

Routine periodontal screening in general dental practice may or may not be conducted depending on various factors including the requirements of the regulatory bodies in the countries where the studies were conducted. In a study in Saudi Arabia, only 36% of the GDPs reported performing periodontal screening routinely [15] while higher percentages of 87% and 95% have been reported in an Australian study [16] and 71.4% among GDPs in Scotland [17]. Locally, only 55.6% of government dentists claimed that they screened for periodontal diseases on all new patients [18]. The lack of emphasis for routine periodontal screening as well as scarcity of periodontal probes were the reasons reported for the lower screening levels in Malaysia.

To overcome the challenges in periodontal screening during daily clinical practice, alternative methods of screening have been developed. These include self-reported periodontal screening questions [19], salivary levels of haemoglobin [20], active MMP-8 detected in GCF [21], and salivary peptide biomarkers [22]. Digital assessment methods are less investigated with limited published literature available. However, these alternative methods have yet to gain popularity in clinical practice, possibly due to the additional training and armamentarium needed to apply them [23].

The module in this study was developed with the aim of helping general dental practitioners conduct periodontal disease screening in a faster and more comprehensive way. The module, which is in digital form, provides more components of examination that can help dental practitioners make faster clinical decisions and broadly categorise patients into different case definitions of periodontal disease. These broad categories will enable the practitioners to decide on the next step of the assessment, the appropriate treatment plan, or the need for referral depending on the clinical situation.

The clinical components of this module are not as extensive as the traditional 6-point periodontal charting. The clinical components assessed are those typically assessed by GDPs in their practice and can potentially assist the GDPs to structure their assessment using the different steps and rows. The module is also aligned with the most recent guidelines for the classification of periodontal disease [11]. Other periodontal screening tools available for this population are in the form a questionnaire and designed for self-assessment by the patients rather than for use by GDPs [12].

In this current study, 12 GDPs were invited to participate in the validation of the module. The calculated IRR gave values ranging from 62.5% to 100.0%, which indicates an acceptable result [24]. This gives an indication that the module can be used by GDPs with a high degree of agreement among independent clinicians with very little additional training, all of which can be conducted remotely using an online platform. Other studies on remote clinical consultation also reports on the feasibility of identifying a generic minimum data set for specific consultation processes [25]. The clinical data set in the newly introduced module can hence be used as a basis for future studies on the feasibility of remote training for periodontal screening and consultation.

Limitations of this study include the convenience sampling method used for both phases of validation, which may lead to sampling bias. The selection of patients for validation by the GDPs may not have included a range of different clinical cases, limiting the applicability of the validation. While a distribution of patients from all three categories of Periodontitis Uncontrolled, Periodontitis Stable, and Periodontitis Remission were assessed in the validation, only four Periodontitis Remission cases were included. In addition, a diagnosis statement of periodontal status cannot be given based on this assessment module because no intraoral periapical radiograph was taken during the assessment, hence staging and grading of periodontal disease cannot be assessed [26]. Furthermore, the number of calibrations performed was only twice on each GDP, which could affect the reliability of the percentage agreement reported in this study. Nevertheless, the attempt made in this study may provide a basis for effort in promoting the identification of periodontitis at large in general dental practices.

## 5. Conclusions

The new online self-guided periodontal health screening module was highly accepted by graduating dental trainees and private general dental practitioners for its ease of understanding and technique, design, and perceived benefits for faster and better decision-making. Within the limits of this study, we believe that the development of the module is befitting and necessary for ease of periodontitis identification at general dental practices. The new module showed promising results for acceptance and application in general dental clinics.

## Figures and Tables

**Table 1:** Undergraduates’ characteristics, n = 156.

Characteristics of Participants	n (%)
**Gender:**	
Male	43 (27.6)
Female	113 (72.4)
**Current tool used to assess periodontal health:**	
BPE—Basic Periodontal Examination	106 (67.9)
CPITN—Community Periodontal Index & Treatment Needs	39 (25.0)
Full clinical assessment	11 (7.1)

**Table 2:** Students’ feedback on their current periodontal assessment skills.

	Difficulty Level				
Skills:	Very Difficult	Difficult	Moderate	Easy	Very Easy
Diagnosing patients	6 (3.9)	10 (6.4)	30 (19.2)	64 (41.0)	46 (29.5)
Managing patients	17 (10.9)	65 (35.9)	49 (31.4)	28 (17.9)	6 (3.9)

**Table 3:** Students’ perceived competency and acceptance of using the new assessment tool.

	**Perceived Competency Level**				
**Perceived Competency Skills in:**	**Very Poor**	**Poor**	**Satisfactory**	**Good**	**Very Good**
diagnosing patients	3 (1.9)	16 (10.3)	37 (23.7)	54 (34.6)	46 (29.5)
understanding of contents	0	1 (0.6)	29 (18.6)	83 (53.2)	43 (27.6)
understanding the design	0	3 (1.9)	18 (11.5)	72 (46.2)	63 (40.4)
	**Agreement**				
**This new tool could help me:**	**Strongly Disagree**	**Disagree**	**Somewhat Agree**	**Agree**	**Strongly Agree**
screen all my new patients in the clinic anytime.	1 (0.6)	4 (2.6)	22 (14.1)	63 (40.4)	66 (42.3)
screen all my patients faster.	2 (1.3)	12 (7.7)	29 (18.6)	62 (39.7)	51 (32.7)
make beneficial decisions faster for the management of my patients.	1 (0.6)	2 (1.3)	12 (7.7)	74 (47.4)	67 (43.0)

**Table 4:** Socio-demographics of the GDPs who participated in the study, n = 12.

Characteristics	n (%)
Age (years)	36.17 ± 9.91 range 27–52 years, median 32
Gender	
Male	3 (25%)
Female	9 (75%
Ethnicity	
Malay	10 (83.3%)
Chinese	2 (16.7%)
Duration of practice (years)	11.67 ± 8.69 range 3–27 years, median 7.5

**Table 5:** Inter Rate Reliability (IRR) of Periodontitis Case Definition between the study participants and the principal investigator using the newly developed module.

GDPs	Patients	Case Defined by GDPs	Case Defined by PI	Inter-Rater Reliability (IRR)/Agreement
Dentist a	Patient 1	PU	PU	100.0%
	Patient 2	PS	PS	100.0%
Dentist b	Patient 3	PS	PS	87.5%
	Patient 4	PR	PR	87.5%
Dentist c	Patient 5	PU	PU	100.0
	Patient 6	PS	PS	87.5%
Dentist d	Patient 7	PU	PU	100%
	Patient 8	PU	PU	87.5%
Dentist e	Patient 9	PS	PS	100.0%
	Patient 10	PU	PU	100.0%
Dentist f	Patient 11	PR	PR	75%
	Patient 12	PU	PU	87.5%
Dentist g	Patient 13	PU	PU	87.5%
	Patient 14	PU	PU	100.0%
Dentist h	Patient 15	PU	PU	87.5%
	Patient 16	PU	PR	62.5%
Dentist i	Patient 17	PS	PS	75.0%
	Patient 18	PS	PS	87.5%
Dentist j	Patient 19	PU	PU	87.5%
	Patient 20	PU	PU	100.0%
Dentist k	Patient 21	PS	PS	87.5
	Patient 22	PU	PU	75.0%
Dentist l	Patient 23	PR	PR	87.5%
	Patient 24	PU	PU	100.0%

PI: Principal Investigator, PU: Periodontitis Uncontrolled, PR: Periodontitis in Remission, PS: Periodontitis Stable.

## Data Availability

Data available on request due to restrictions. The data presented in this study are available on request from the corresponding author. The data are not publicly available due to confidentiality issues.

## Supplementary Materials

The following supporting information can be downloaded at: https://www.mdpi.com/article/10.3390/healthcare10101916/s1, Supplementary S1: Steps of the Module.
