# Supplementary material for: Validation of a Simplified Digital Periodontal Health Screening Module for General Dental Practitioners
Source: Healthcare (Basel). 2022 Sep 30;10(10):1916. doi: 10.3390/healthcare10101916 (PMC9601548; doi:10.3390/healthcare10101916)
Supplement: Supplementary file 1 [file healthcare-10-01916-s001.zip › healthcare-1909142-supplementary.pdf]

**Supplementary S1: Steps of the Module**

The newly developed module has four steps as explained below:

The Step 1 component consists of history taking including the history of periodontitis such as smoking status, gum bleeding status, history of gingival recession, and being told of getting gum treatment. Established systemic conditions of patients such as diabetes mellitus, hypertension, hypercholesterolemia, obesity, and other immuno-deficiency conditions are also recorded.

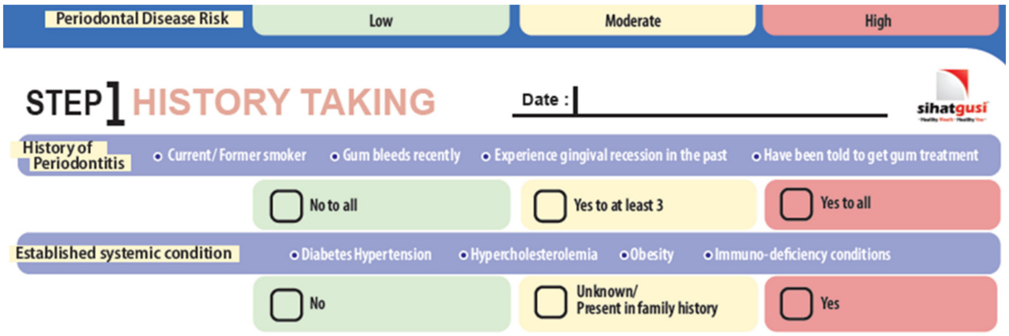


Step 2 comprises a clinical assessment including signs of gingival inflammation, BPE Score, and clinical attachment loss as well as decision making based on the findings. The clinical assessment for clinical attachment loss is based on the interdental recession as observed clinically.

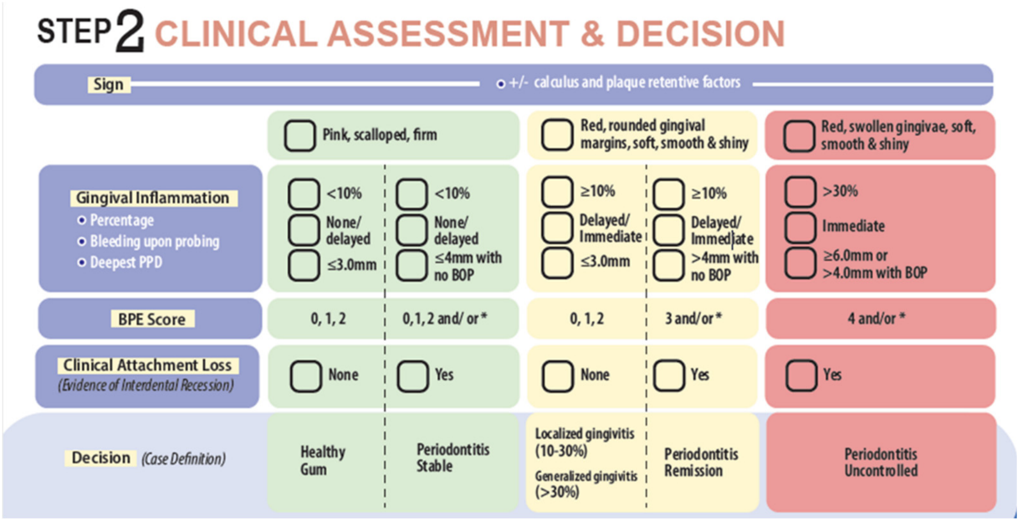


Step 3 is professional management and explanation of treatment procedures. This includes the recommended management of routine maintenance, attention needed or referral to periodontal specialist.

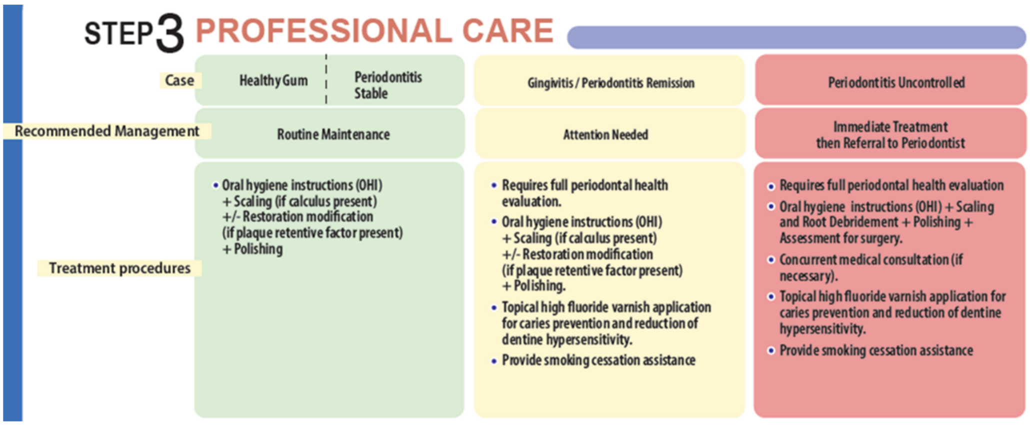


Step 4 is patient self- care recommendations. This includes frequency of dental check-ups, type of homecare recommended and healthy lifestyle advice.

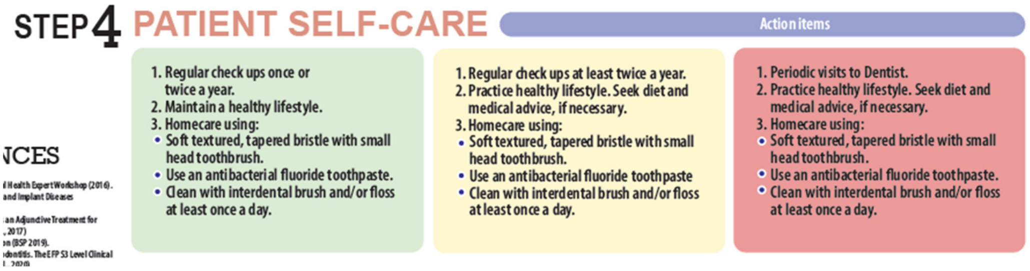


All these components are divided into 3 periodontal disease risks and are indicated by traffic light colour, which is the green colour for low risk, yellow colour for moderate risk, and red colour for high risk.

**Case Scenario**

Three clinical cases are included in the digital case scenario. The details of the cases included clinical photographs (frontal and occlusal view), patient history and clinical features/ findings. These details are those that would normally be available after a routine clinical examination by the general dental practitioner.

Case 1 is a of a 32-year-old female with some gingival recession due to vigorous toothbrushing, minimal bleeding on probing, minimal calculus deposits and has a highest BPE of 2. The case definition for the case is healthy gums. The recommended management is routine maintenance.

Case 2 is a of a fit and healthy 46-year-old male with interdental gingival recession, immediate bleeding on probing noted at 30% of all sites, generalized supra and subgingival calculus and a highest BPE of 3 with the maximum pocket depth of 5 mm. The case diagnosis is periodontitis (remission). The recommended management is ‘attention needed’.

Case 3 is a of a 21-year-old male with no known medical illness. He presents with interdental gingival recession, immediate bleeding on probing noted at 50% of the sites, a highest BPE of 4* and the

maximum pocket depth of 7 mm. The case diagnosis is periodontitis (uncontrolled). The recommended management is immediate treatment and referral to periodontal specialist.
